# Plasma adiponectin levels predict cognitive decline and cortical thinning in mild cognitive impairment with beta-amyloid pathology

**DOI:** 10.1186/s13195-022-01107-3

**Published:** 2022-11-04

**Authors:** Keun You Kim, Junghee Ha, Minae Kim, So Yeon Cho, Hyunjeong Kim, Eosu Kim, for the Alzheimer’s Disease Neuroimaging Initiative

**Affiliations:** 1 Department of Psychiatry, Institute of Behavioral Science in Medicine, Yonsei University College of Medicine, 50-1 Yonsei-ro, Seodaemun-gu, Seoul, Republic of Korea; 2 Department of Psychiatry, Seoul Metropolitan Government - Seoul National University Boramae Medical Center, Seoul, Republic of Korea; 3 Brain Korea 21 FOUR Project for Medical Science, Yonsei University College of Medicine, Seoul, Republic of Korea

**Keywords:** Adiponectin, Alzheimer’s disease, Beta-amyloid, Cortical thickness, Leptin, Mild cognitive impairment

## Abstract

**Background:**

Blood adiponectin and leptin are adipokines that emerged as potential biomarkers for predicting Alzheimer’s disease (AD) owing to their strong connection with obesity. Although obesity affects the relation between beta-amyloid (Aβ) aggregation and cognitive decline, the longitudinal interactive effect of adipokines and Aβ on cognition and brain structures in humans remains unexplored. Hence, we investigated whether plasma levels of adiponectin and leptin are associated with future cognitive decline and cortical thinning across Aβ conditions (Aβ [+] and Aβ [−]) in individuals with mild cognitive impairment (MCI).

**Methods:**

Of 156 participants with MCI from the longitudinal cohort study of Alzheimer’s Disease Neuroimaging Initiative (ADNI), 31 were Aβ (−) and 125 were Aβ (+) as determined by CSF analysis. The Alzheimer’s Disease Assessment Scale-Cognitive Subscale (ADAS-Cog) scores and the thickness of the parahippocampal and entorhinal cortices were used to evaluate cognition and brain structure, respectively. After stratifying groups by Aβ conditions, the association of cognitive and brain structural changes with baseline plasma levels of adiponectin and leptin was examined.

**Results:**

Of the total 156 participants, 51 were women (32.7%). The mean age of participants was 74.5 (standard deviation 7.57), and the mean follow-up period was 54.3 months, without a difference between the Aβ (+) and (−) groups. After adjustment for confounders, higher plasma adiponectin levels were associated with a faster increase in ADAS-Cog scores, indicating faster cognitive decline under the Aβ (+) condition (beta = 0.224, *p* = 0.018). Likewise, participants with higher plasma adiponectin presented faster cortical thinning in the bilateral parahippocampal cortices under the Aβ (+) condition (beta = − 0.004, *p* = 0.012 for the right side; beta = − 0.004, *p* = 0.025 for the left side). Interestingly, plasma adiponectin levels were not associated with longitudinal ADAS-Cog scores or cortical thickness in the Aβ (−) condition. Plasma leptin levels were not predictive of cognition or cortical thickness regardless of Aβ status.

**Conclusion:**

Plasma adiponectin can be a potential biomarker for predicting the speed of AD progression in individuals with Aβ (+) MCI.

**Supplementary Information:**

The online version contains supplementary material available at 10.1186/s13195-022-01107-3.

## Background

Prediction of Alzheimer’s disease (AD) in the preclinical stage could be facilitated by detecting the presence of beta-amyloid (Aβ) pathology through cerebrospinal fluid (CSF) analysis or positron emission tomography (PET) [1, 2]. However, given the multiple factors contributing to AD pathogenesis, cognitive decline does not solely depend on the degree of Aβ pathology. Furthermore, the range of Aβ deposition in individuals with preclinical AD overlaps with that in normal individuals [1, 2]. This suggests that additional biomarkers are required to define preclinical AD more reliably.

Obesity is regarded as a risk factor for AD [3]. Hormones secreted from adipose tissue, referred to as adipokines, have been found to affect brain function and AD pathology, as well as to regulate energy expenditure and food intake [3]. Adiponectin and leptin, the two most extensively studied adipokines, have been reported to have neuroprotective effects and to be positively correlated with memory function [4]. Thus, they have emerged as potential supplementary biomarkers to Aβ for detecting early cognitive decline [4–7].

However, prior studies evaluating the predictive power of adipokines for cognitive decline or risk of AD have shown inconsistent results [5–18], and none of them has considered the status of Aβ pathology (Table 1). Given the previous in vitro findings that adiponectin and leptin have a role against Aβ metabolism [19, 20], the association of adipokines with cognitive decline might be also dependent on the presence of Aβ pathology. This possibility is supported by a recent finding that obesity aggravates cognitive decline in the presence of Aβ pathology even in individuals without dementia [21].

**Table 1:** Characteristics of previous studies of the relationship between adiponectin or leptin and cognitive decline or risk of dementia

First author, year	Study design	Follow-up years	Sample size (*n*)	Age (years)	Female (%)	Results
**Adiponectin**						
van Himbergen, 2012 [6]	Prospective cohort study	13	840	72.6	64.4	**Positive association:** Higher plasma adiponectin levels were associated with a higher risk of incident all-cause dementia and AD only in females, not in males.
Teixeira, 2013 [8]	Cross-sectional/prospective cohort study	2.5	157	71.4	73.6	(Cross-sectional study) **Negative association:** Serum adiponectin levels were lower in MCI and AD as compared to normal cognition. (Prospective study) **No association** between adiponectin levels and incident MCI or AD.
Kitagawa, 2016 [9]	Prospective cohort study	6.9	466	67.8	43	**No association** between serum adiponectin levels and incident all-cause dementia, AD, and vascular dementia.
Gilbert, 2018 [10]	Prospective cohort study	1.2	205	80.6	65.4	**No association** between baseline serum adiponectin levels and the course of cognitive decline.
van Andel, 2021 [7]	Prospective cohort study	3	898	69.8	53.9	**Positive association:** Higher serum adiponectin levels were associated with more decline in general cognitive function and information processing speed only in females.
Kim, 2021 [11]	Prospective cohort study	5.3	345	76.6	34.5	**No association** between baseline serum adiponectin levels and the course of cognitive decline in MCI.
Ban, 2007 [12]	Cross-sectional study	0	60	79.3	45	**No association:** Serum adiponectin levels were not significantly different between normal cognition and vascular dementia group.
Une, 2011 [13]	Cross-sectional study	0	73	74.7	60.3	**Positive association:** Plasma adiponectin levels were higher in MCI and AD as compared to normal cognition.
Letra, 2019 [18]	Cross-sectional study	0	124	73.9	68.5	**Positive association:** Plasma adiponectin levels were higher in AD as compared to MCI.
**Leptin**						
Lieb, 2009 [5]	Prospective cohort study	8.3	785	79	62	**Negative association:** Higher plasma leptin levels were associated with a lower risk of incident all-cause dementia and AD.
Holden, 2009 [14]	Prospective cohort study	5	2870	73.7	51	**Negative association:** Higher serum leptin levels were associated with less cognitive decline.
Gustafson, 2012 [15]	Prospective cohort study	32	1384	46.9	100	**No association** between leptin levels and incident all-cause dementia.
Zeki Al Hazzouri, 2013 [16]	Prospective cohort study	4	579	82.6	100	**Negative association:** Higher leptin levels were associated with a lower risk of incident all-cause dementia or MCI only in those with BMI < 25 kg/m^2^.
Oania, 2015 [17]	Prospective cohort study	3	352	74.7	36.4	**No association** between leptin levels and incident all-cause dementia.
Gilbert, 2018 [10]	Prospective cohort study	1.2	205	80.6	65.4	**No association** between baseline serum leptin levels and cognitive decline.
van Andel, 2021 [7]	Prospective cohort study	3	898	69.8	53.9	**No association** between baseline serum leptin levels and cognitive decline.
Ban, 2007 [12]	Cross-sectional study	0	60	79.3	45	**No association:** Serum leptin levels were not significantly different between normal cognition and vascular dementia group.
Letra, 2019 [18]	Cross-sectional study	0	124	73.9	68.5	**No association:** Plasma leptin levels were not significantly different between MCI and AD.

Note that all the studies listed here did not consider the status of Aβ pathology

*Abbreviations*: *Aβ* amyloid-β, *AD,* Alzheimer’s disease, *BMI*, body mass index, *MCI* mild cognitive impairment

Hence, in this study, we investigated whether the association of adipokines with cognitive decline is affected by the presence or absence of Aβ pathology in individuals with mild cognitive impairment (MCI), who are considered to be at high risk for AD [22]. To validate the effects of adipokines on cognition, we also analyzed cortical thickness as measured by magnetic resonance imaging (MRI) scans.

## Methods

### Participants and data acquisition

The data used in this study was obtained from the Alzheimer’s Disease Neuroimaging Initiative (ADNI) database (http://adni.loni.usc.edu) on November 16, 2020. The ADNI is a multisite longitudinal study performed across 63 sites in the USA and Canada since 2003. One of the goals of the ADNI is to detect AD in the pre-dementia stage using neuroimages and biomarkers (for up-to-date information, see www.adni-info.org). Detailed characteristics of participants including the inclusion and exclusion criteria were described elsewhere [23]. Participants were aged between 55 and 90, fluent in English or Spanish, without stroke (Hachinski Ischemic Score ≤ 4) and major depression (Geriatric Depression Scale score < 6), and with adequate visual and auditory acuity to allow neuropsychological testing. They should not have significant major neurological or psychiatric diseases, such as Parkinson’s disease, stroke, history of alcohol/substance dependence within the past 2 years, or history of schizophrenia or bipolar disorder. Participants with all the following criteria were considered to have MCI [23]: (i) the Mini-Mental State Examination score ≥ 24; (ii) the global Clinical Dementia Rating score = 0.5 with memory box score ≥ 0.5; (iii) the Logical Memory II subscale score, from the Wechsler Memory Scale–Revised (the maximum score of 25), ≤ 8 for 16 years of education, ≤ 4 for 8–15 years of education, and ≤ 2 for 0–7 years of education; and (iv) intact general cognition and functional performance sufficiently preserved such that AD diagnosis cannot be made [22].

Demographic, diagnostic, and cognitive test data were collected from the file named “ADNIMERGE.csv.” While the ADNI project enrolled participants during overall 4 phases (ADNI 1, ADNI GO, ADNI 2, and ADNI 3), all the participants in this study were those who had been initially recruited at the ADNI 1 phase, the period of 5 years since 2004. Only during this ADNI 1 phase, adiponectin and leptin assays were measured. Of a total of 396 ADNI 1 participants with CSF Aβ at baseline, we included those diagnosed with MCI at baseline (*n* = 187). From this eligible population (*n* = 187), 1 participant was excluded owing to the non-availability of plasma adiponectin and leptin. In addition, 30 obese participants with a body mass index (BMI) of 30 kg/m^2^ or over were excluded to avoid potential confounding effects of adiposity. Finally, 156 participants were included in the analysis (Fig. S1). The Institutional Review Board from each participating institution approved this study with written informed consent obtained from all participants.

### Cognitive function assessment

To assess cognitive function, we used the results of the Alzheimer’s Disease Assessment Scale-Cognitive subscale (ADAS-Cog) scores. The standard ADAS-Cog scale includes 11 items measuring memory, language, and praxis. The total score of ADAS-Cog ranges from 0 to 70. Since ADAS-Cog measures the sum of incorrect answers, a higher score indicates a poorer cognitive function. ADAS-Cog scores were measured at baseline and repeated every 6 or 12 months to evaluate the trajectory of cognitive decline.

### Structural brain MRI analysis

Standardized MRI acquisition and preprocessing protocols are described elsewhere (http://adni.loni.usc.edu/methods/mri-tool/mri-analysis/). Imaging data was processed by the team from the University of California at San Francisco, who performed cortical reconstruction and volumetric segmentations with the FreeSurfer version 4.3 from 1.5-T T1 MRI images (http://surfer.nmr.mgh.harvard.edu/). We set regions of interest (ROIs) in the parahippocampal cortex (PHC) and entorhinal cortex (ERC), which are key areas of cognition and AD pathology [24, 25]. The cortical thickness values of the ROIs were used in the analyses. Same as ADAS-Cog, MRI scans were conducted every 6 or 12 months in each participant.

### Biomarker measurements

Between different isoforms of Aβ with an amino acid length of 42 and 40 (Aβ 1–42 and Aβ 1–40, respectively), we used data of Aβ 1–42 which is the core pathology of AD [1]. CSF Aβ was measured using the fully automated Roche Elecsys® immunoassay, which is known to be more accurate than other methods [26]. CSF immunoassays were conducted at the ADNI Biomarker Laboratory, University of Pennsylvania, according to the preliminary kit manufacturer’s instructions and as described in previous studies [26]. We determined Aβ pathology at baseline by CSF Aβ levels referred by previously proposed thresholds: CSF Aβ ≥ 1100 pg/mL for negative pathology (Aβ [−]) and < 1100 for positive pathology (Aβ [+]) [27].

We used data of plasma adiponectin and leptin from the Biomarkers Consortium Project “Use of Targeted Multiplex Proteomic Strategies to Identify Plasma-Based Biomarkers in Alzheimer’s Disease.” Plasma samples were obtained in EDTA tubes after overnight fasting and frozen within 120 min. Plasma adipokines were analyzed by a multiplex immunoassay panel based on the Luminex xMAP platform provided by Rules-Based Medicine (RBM, Austin, TX, USA). The Luminex xMAP technology is an immunological method quantifying multiple target proteins simultaneously through detecting fluorescent microspheres. Details of the quantification methods are available at http://adni.loni.usc.edu/wp-content/uploads/2010/11/BC_Plasma_Proteomics_Data_Primer.pdf. For quality control, plasma levels of adiponectin and leptin were log-transformed owing to non-normal distribution.

### Other clinical variables affecting the levels of adipokines or the risk of AD

The presence of hypertension, relevant to both adipokines and AD [4], was determined as one of the followings: (i) taking antihypertensive medications, (ii) systolic blood pressure ≥ 140 mmHg, or (iii) diastolic blood pressure ≥ 90 mmHg. Given that the levels of adipokines could be affected by the status of glucose homeostasis [28] and renal failure [29], the presence of diabetes mellitus, levels of plasma insulin, and estimated glomerular filtration rate (eGFR) were also examined in this study. Based on the American Diabetes Association guideline [30], the presence of diabetes mellitus was defined by fasting blood glucose ≥ 126 mg/dL or taking glucose-lowering agents. Since peroxisome proliferator-activated receptor γ (PPARγ) agonists were glucose-lowering agents affecting the levels of adiponectin [31], we investigated whether participants were taking PPARγ agonists such as pioglitazone and rosiglitazone. However, as only one participant out of 156 was identified to be on rosiglitazone medication, we did not consider the adjustment with this information. Additionally, a history of ever smoking and alcohol abuse was also considered covariates in the analyses.

### Statistical analysis

The baseline demographics and clinical characteristics in strata of Aβ status were compared using Student’s *t*-test for continuous variables and *χ*^2^ tests for categorical variables. For identifying the variables associated with baseline plasma adipokines, we conducted multiple regression analyses in which the levels of baseline adiponectin and leptin were the outcome variables after adjustment for age, sex, years of education, number of apolipoprotein (APOE) ε4 genotype, BMI, and history of hypertension, diabetes mellitus, smoking and alcohol abuse, level of plasma insulin, and eGFR.

To test whether the longitudinal effect of adipokines on cognition and cortical thickness is influenced by Aβ pathology, linear mixed-effect models were applied with participants stratified by Aβ status. The fixed effects were the plasma levels of adipokines (adiponectin and leptin), time since baseline, and the interaction term of adipokine × time. Random effects included slope and intercept. The outcome variables were ADAS-Cog score and thickness in the bilateral PHC and ERC. Covariates included baseline age, sex, years of education, number of APOE ε4 genotype, BMI, and history of hypertension, diabetes mellitus, smoking and alcohol abuse, level of plasma insulin, and eGFR. In case of a significant predictive effect of the plasma adiponectin or leptin on cognitive decline, we examined additional linear mixed-effect models where the BMI change was an outcome variable instead of the ADAS-Cog score. To test whether the association between longitudinal cognition and baseline adipokine levels would vary by sex, we conducted a subgroup analysis stratified by sex with ADAS-Cog score as an outcome variable. Additionally, extra linear mixed-effect models were performed where the interaction term of plasma adipokine (adiponectin or leptin) level × time since baseline × sex were included. Missing values were addressed by listwise deletion.

All statistical analyses were conducted using R, version 4.0.5, and the lme4 package, version 1.1–26 was used for fitting linear mixed-effect models [32] with statistical significance set at alpha = 0.05.

## Results

### Baseline characteristics of participants

The baseline demographic and clinical characteristics of the participants dichotomized by Aβ pathology are shown in Table 2. Fifty-one (32.7%) participants were women, the mean age at baseline was 74.5 (SD 7.57), and the mean follow-up period was 54.3 months (SD 38.4) without a difference between the Aβ (+) and Aβ (−) groups. Compared with participants with Aβ (−), those with Aβ (+) showed higher mean ADAS-Cog score and ratio of APOE ε4 (+) and lower mean thickness of the right ERC. Notably, plasma adiponectin and leptin levels were comparable between the two groups. The numbers of participants who underwent ADAS-Cog and MRI scanning at each time point are described in Additional file 1: Table S1, and the numbers and reasons of non-participation for fully withdrawn participants are reported in Additional file 1: Table S2.

**Table 2:** Demographic and clinical characteristics of the study groups at baseline

	Aβ (−) (*n* = 31)	Aβ (+) (*n* = 125)	*p*-value
Age (years)	75.3 (8.59)	74.3 (7.31)	0.542
Sex			
Male	21 (67.7%)	84 (67.2%)	1
Female	10 (32.3%)	41 (32.8%)	
Education (years)	15.6 (3.32)	16.1 (2.85)	0.461
History of hypertension			
No	12 (38.7%)	50 (40.0%)	1
Yes	19 (61.3%)	75 (60.0%)	
History of diabetes mellitus			
No	26 (83.9%)	110 (88.0%)	0.752
Yes	5 (16.1%)	15 (12.0%)	
PPARγ agonists use^a^			
No	31 (100%)	124 (99.2%)	1
Yes	0 (0%)	1 (0.8%)	
Ever smoker			
No	15 (48.4%)	75 (60.0%)	0.333
Yes	16 (51.6%)	50 (40.0%)	
History of alcohol abuse			
No	28 (90.3%)	119 (95.2%)	0.540
Yes	3 (9.7%)	6 (4.8%)	
BMI (kg/m^2^)	25.1 (2.50)	24.6 (2.51)	0.321
Plasma insulin (uIU/mL)	3.12 (3.37)	2.32 (2.79)	0.227
eGFR (mL/min/1.73 m^2^)	69.4 (16.3)	69.8 (15.0)	0.907
Plasma adiponectin (μg/mL)	6.33 (3.70)	6.77 (3.95)	0.564
Plasma leptin (ng/mL)	10.5 (9.79)	9.61 (9.29)	0.632
CSF Aβ (pg/mL)^b^	1530 (181)	625 (182)	< 0.001
Number of APOE ε4 allele			
0	26 (83.9%)	47 (37.6%)	< 0.001
1	5 (16.1%)	61 (48.8%)	
2	0 (0%)	17 (13.6%)	
ADAS-Cog	9.34 (3.93)	12.4 (4.73)	< 0.001
Cortical thickness in PHC, right (mm)	2.46 (0.340)	2.34 (0.317)	0.097
Cortical thickness in PHC, left (mm)	2.49 (0.334)	2.37 (0.367)	0.089
Cortical thickness in ERC, right (mm)	3.31 (0.468)	3.11 (0.502)	0.041
Cortical thickness in ERC, left (mm)	3.04 (0.554)	2.98 (0.510)	0.606
Follow-up period (months)	56.7 (41.7)	53.7 (37.6)	0.713
Number of repeated measurements			
ADAS-Cog	7.06 (3.48)	6.44 (2.78)	0.359
MRI	5.26 (1.75)	5.34 (2.04)	0.814

Data are presented as mean (standard deviation) for continuous variables and *n* (%) for categorical variables

*Abbreviations*: *Aβ* amyloid-β, *ADAS-Cog* Alzheimer’s Disease Assessment Scale-Cognitive subscale, *APOE* apolipoprotein E, *BMI* body mass index, *CSF* cerebrospinal fluid, *ERC* entorhinal cortex, *eGFR* estimated glomerular filtration rate, *MRI* magnetic resonance imaging, *PHC* parahippocampal cortex, *PPARγ* peroxisome proliferator-activated receptor γ, *SE* standard error

^a^PPARγ agonists investigated were pioglitazone and rosiglitazone

^b^Because of the upper technical limit of measuring range, the levels of 1700 pg/mL or more were treated as 1700 pg/mL. The levels under 1100 pg/mL were determined as Aβ (+)

### Relationship between adipokines and clinical characteristics at baseline

The results of cross-sectional multiple regression analyses at baseline are shown in Table 3. Consistent with previous findings [6, 7], the levels of plasma adiponectin and leptin were elevated in female participants. At baseline, BMIs and the levels of plasma insulin were negatively associated with the levels of plasma adiponectin but positively associated with those of leptin, which are also consistent with the results from previous studies [4, 28]. Cortical thickness was not associated with baseline plasma adiponectin or leptin levels except for thickness in the left PHC, which was inversely associated with plasma adiponectin levels. Of note, the levels of adiponectin or leptin were not associated with Aβ status and ADAS-Cog scores in these cross-sectional analyses.

**Table 3:** Results of the cross-sectional multiple regression model predicting adipokine levels at baseline

Predictor	Outcome—adiponectin			Outcome—leptin		
	Beta	SE	*p*-value	Beta	SE	*p*-value
Intercept	1.480	0.491	0.003	− 0.952	0.587	0.107
Aβ (+)	− 0.028	0.050	0.580	0.059	0.060	0.333
ADAS-Cog	0.007	0.005	0.158	0.006	0.006	0.310
PHC, right	0.115	0.081	0.160	− 0.014	0.097	0.884
PHC, left	− 0.263	0.076	< 0.001	− 0.007	0.090	0.934
ERC, right	0.003	0.051	0.958	− 0.062	0.061	0.311
ERC, left	0.069	0.051	0.176	0.016	0.061	0.796
Age	0.000	0.003	0.935	− 0.001	0.003	0.806
Sex (female)	0.125	0.043	0.004	0.481	0.051	< 0.001
Education	− 0.003	0.007	0.707	0.000	0.008	0.966
APOE ε4	0.009	0.029	0.762	− 0.038	0.035	0.286
BMI	− 0.025	0.008	0.001	0.074	0.009	< 0.001
Hypertension	− 0.004	0.037	0.920	0.088	0.045	0.050
Diabetes mellitus	− 0.037	0.057	0.518	− 0.110	0.069	0.110
PPARγ agonist	0.306	0.232	0.190	− 0.127	0.278	0.647
Smoking	0.050	0.040	0.219	0.046	0.048	0.337
Alcohol	− 0.015	0.083	0.858	− 0.047	0.100	0.637
Insulin	− 0.132	0.060	0.030	0.332	0.072	< 0.001
eGFR	0.000	0.001	0.832	− 0.002	0.002	0.123

The levels of plasma adiponectin, leptin, and insulin were log-transformed

*Abbreviations*: *Aβ* amyloid-β, *ADAS-Cog* Alzheimer’s Disease Assessment Scale-Cognitive subscale, *APOE* apolipoprotein E, *BMI* body mass index, *ERC* entorhinal cortex, *eGFR*, estimated glomerular filtration rate, *PHC* parahippocampal cortex, *PPARγ* peroxisome proliferator-activated receptor γ, *SE* standard error

### The impacts of baseline adipokines on changes in cognition and cortical thickness across Aβ conditions

#### Adiponectin

In participants with Aβ (+), the linear mixed-effect models revealed statistically significant effects of two-way interactions between plasma adiponectin levels and time on ADAS-Cog score (beta = 0.224, *p* = 0.018) and thickness in the bilateral PHC (beta = − 0.004, *p* = 0.012 for the right side; beta = − 0.004, *p* = 0.025 for the left side; left panel of Table 4 and Additional file 1: Table S3). However, the same linear mixed-effect models failed to find significance in participants with Aβ (−) (right panel of Table 4 and Additional file 1: Table S3). These results indicate that the rate of changes in cognition and cortical thickness are significantly dependent on the baseline levels of plasma adiponectin exclusively in the Aβ (+) condition. After finding the longitudinal effect of adiponectin on cognitive decline and cortical thinning, we performed an additional linear mixed-effect model where the BMI change was an outcome variable (Table 4 and Additional file 1: Table S3). In both Aβ (+) and Aβ (−) groups, the baseline levels of plasma adiponectin were not associated with prospective BMI changes. The exclusion of the PPARγ agonist user did not change these results of linear mixed-effect models (Additional file 1: Table S4).

**Table 4:** Predictive effect of the interaction between baseline adiponectin and time since baseline

Outcome	Adiponectin × time interaction					
	Aβ (+)			Aβ (−)		
	Beta	SE	*p*-value	Beta	SE	*p*-value
ADAS-Cog	0.224	0.093	0.018	− 0.018	0.054	0.744
PHC, right	− 0.004	0.002	0.012	− 0.002	0.002	0.529
PHC, left	− 0.004	0.002	0.025	− 0.002	0.003	0.411
ERC, right	− 0.007	0.003	0.024	0.010	0.005	0.042
ERC, left	− 0.005	0.003	0.122	0.002	0.004	0.690
BMI change	− 0.004	0.016	0.805	0.054	0.042	0.216

Models were adjusted for the following covariates: baseline age, sex, years of education, number of APOE ε4 genotype, BMI, history of hypertension, diabetes mellitus, smoking and alcohol abuse, the levels of plasma insulin, and eGFR. The levels of plasma adiponectin and insulin levels were log-transformed

*Abbreviations*: *Aβ* amyloid-β, *ADAS-Cog* Alzheimer’s Disease Assessment Scale-Cognitive subscale, *APOE* apolipoprotein E, *BMI* body mass index, *ERC* entorhinal cortex, *eGFR* estimated glomerular filtration rate, *PHC* parahippocampal cortex, *SE* standard error

**Fig. 1:**
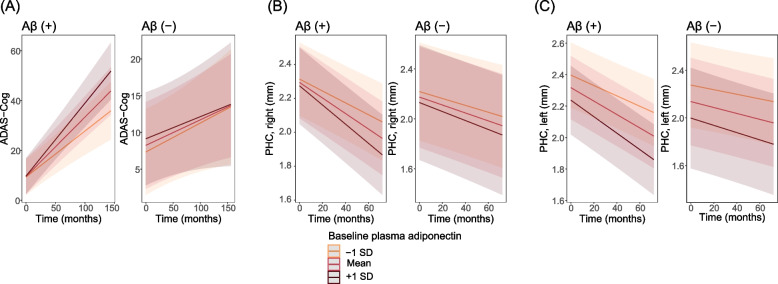
Higher plasma adiponectin levels are associated with faster cognitive decline and cortical thinning in PHC in participants with Aβ (+), but not in those with Aβ (−). The plotted lines are estimated values over time under different conditions of plasma adiponectin (− 1 SD above the mean, mean, and 1 SD below the mean baseline levels). *Abbreviations*: *Aβ* amyloid-β, *ADAS-Cog* Alzheimer’s Disease Assessment Scale-Cognitive subscale, *PHC* parahippocampal cortex, *SD* standard deviation

A detailed examination revealed that in participants with Aβ (+), the rate of change in ADAS-Cog score of those with higher (mean + 1 SD) plasma adiponectin level was 0.292/month (SE = 0.031), which was faster than that of participants with lower (mean – 1 SD) adiponectin levels (0.184/month [SE = 0.032]; Fig. 1A, left panel). Likewise, among participants with Aβ (+), the rate of atrophy in the bilateral PHC was higher in those with higher plasma adiponectin levels than in participants with low adiponectin levels (higher adiponectin level: − 0.0057 mm/month [SE = 0.0006] in the right side and − 0.0052 mm/month [SE = 0.0006] in the left side; lower adiponectin level: − 0.0035 mm/month [SE = 0.0006] in the right side and − 0.0033 mm/month [SE = 0.0006] in the left side; Fig. 1B, C, left panels). On the other hand, among participants with Aβ (−), the longitudinal trends of ADAS-Cog score and PHC thickness were independent of baseline adiponectin levels (Fig. 1A-C, right panels).

#### Leptin

**Fig. 2:**
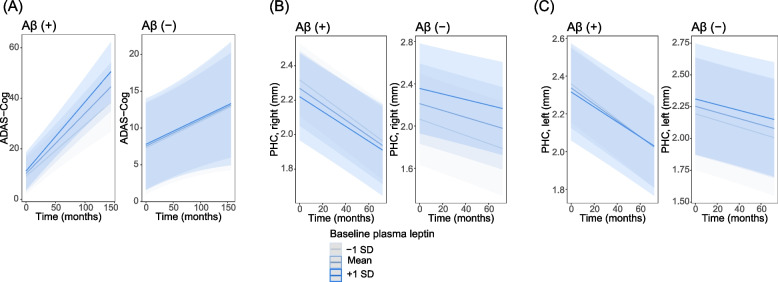
Plasma leptin levels are not associated with cognitive decline and cortical thinning in PHC regardless of Aβ status. The plotted lines are estimated values over time under conditions of plasma leptin (− 1 SD above the mean, mean, and 1 SD below the mean baseline levels). *Abbreviations*: Aβ, amyloid-β; ADAS-Cog, Alzheimer’s Disease Assessment Scale-Cognitive subscale; PHC, parahippocampal cortex; SD, standard deviation

In both the Aβ (+) and Aβ (−) groups, the two-way interaction between plasma leptin and time did not significantly predict the ADAS-Cog score or thickness in the PHC and ERC (Table 5 and Additional file 1: Table S5). These results indicate that age-related cognitive decline and cortical atrophy were not affected by baseline plasma leptin levels regardless of Aβ status. Figure 2A-C depicts that the rate of change in cognition and thickness in PHC was comparable between participants with different baseline leptin levels.

**Table 5:** Predictive effect of the interaction between baseline leptin and time since baseline

Outcome	Leptin × time interaction					
	Aβ (+)			Aβ (−)		
	Beta	SE	*p*-value	Beta	SE	*p*-value
ADAS-Cog	0.091	0.059	0.128	0.000	0.030	0.988
PHC, right	0.001	0.001	0.485	0.001	0.001	0.190
PHC, left	0.001	0.001	0.428	0.000	0.001	0.774
ERC, right	− 0.001	0.002	0.723	0.000	0.002	0.998
ERC, left	0.000	0.002	0.833	0.000	0.002	0.890

Models were adjusted for the following covariates: baseline age, sex, years of education, number of APOE ε4 genotype, BMI, history of hypertension, diabetes mellitus, smoking and alcohol abuse, the levels of plasma insulin, and eGFR. The levels of plasma leptin and insulin levels were log-transformed

*Abbreviations*: *Aβ* amyloid-β, *ADAS-Cog* Alzheimer’s Disease Assessment Sscale-Cognitive subscale, *APOE* apolipoprotein E, *BMI* body mass index, *ERC* entorhinal cortex, *eGFR* estimated glomerular filtration rate, *PHC* parahippocampal cortex, *SE* standard error

#### Effect of sex on the association between adipokines and cognition

After stratifying Aβ (+) and Aβ (−) groups by sex, we found no significant associations between baseline adipokine (adiponectin and leptin) levels and longitudinal ADAS-Cog scores both in male and female groups regardless of Aβ status (Additional file 1: Table S6). Likewise, in the extra linear mixed-effect models, the three-way interaction term of plasma adipokine (adiponectin or leptin) level × time × sex was not significantly associated with prospective ADAS-Cog scores in both the Aβ (+) and Aβ (−) groups (Additional file 1: Table S6). These findings suggest that the relationships between baseline plasma levels of adiponectin or leptin and future cognition were not functions of sex.

## Discussion

We investigated the interactive effect of plasma adiponectin or leptin and Aβ pathology on progressive cognitive decline and cortical thinning in individuals with MCI. Higher plasma adiponectin levels at baseline predicted the faster cognitive decline and cortical thinning in the PHC in participants with Aβ (+) but not in those with Aβ (−). Baseline plasma leptin levels were not significant in predicting cognitive decline and progress in cortical atrophy regardless of Aβ pathology.

### Adiponectin

The cross-sectional regression analysis showed that plasma adiponectin levels were not associated with Aβ status, cognition, and cortical thickness, except in the left PHC (Table 3). Despite previous findings of the neuroprotective role of adiponectin [33] and its potential as a diagnostic biomarker [10], our results suggest that plasma adiponectin level does not reflect the current status of the brain or AD pathology.

Longitudinal trajectory analysis performed by linear mixed-effect models showed that participants with higher levels of plasma adiponectin presented accelerated cognitive decline and cortical thinning only in the Aβ (+) status (Table 4 and Fig. 1). Previous longitudinal studies addressing the question of whether blood levels of adiponectin are associated with the risk of AD have shown mixed results [6–10]. However, these prior studies did not consider the presence or absence of Aβ pathology. Our results indicate that the predictive effect of adiponectin on neurodegeneration of the brain may become greater under Aβ pathology. Prior animal study showing the protective role of adiponectin against Aβ-related neurotoxicity [20] still supports our findings. Given that obesity accelerates memory decline under Aβ pathology [21], our results support the possibility of adiponectin as a potential link between abnormal adiposity and the Aβ-related pathology of AD [34].

Despite the well-established neuroprotective role of adiponectin, our results showed that higher levels of plasma adiponectin predicted faster cognitive decline in the Aβ (+) group, which is consistent with some previous prospective cohort studies [6, 7]. This counterintuitive result, referred to as adiponectin paradox, could be explained by weight loss as a prodromal symptom of AD [4]. Although adiponectin is predominantly produced from adipose tissue, the levels of blood adiponectin are decreased in obesity [35] and increased after weight loss [36], as in our result of the inverse correlation of the levels of plasma adiponectin and BMI (Table 3). Moreover, in our study sample, the levels of baseline plasma adiponectin were not associated with prospective BMI changes, regardless of Aβ conditions (Table 4), consistent with previous findings [37]. These findings suggest that the elevated levels of plasma adiponectin might indicate weight loss seen in the prodromal stage of AD, rather than that adiponectin might contribute to AD pathogenesis. This might explain why elevated plasma adiponectin predicted cognitive decline only in participants with Aβ (+) status. However, several issues remain to be addressed. For instance, it should be clarified if adiponectin is exclusively secreted from adipose tissue: if there have been other unidentified sources of brain adiponectin [4, 34] or different roles depending on target organs (brain vs periphery), plasma levels alone would not reflect the expected (positive) role of adiponectin in the brain. In addition, different molecular forms of adiponectin and ratios between them could be another factor contributing to the paradoxical findings.

### Leptin

As in the case of adiponectin, we found no difference in the baseline plasma leptin levels between the Aβ (+) and Aβ (−) groups in participants with MCI (Table 2). In accordance with previous studies showing that blood leptin levels are not different between individuals with and without dementia [12, 18], our findings suggest that blood leptin, as well as adiponectin, does not seem to sensitively reflect the current disease state.

In contrast to adiponectin, baseline leptin levels did not predict longitudinal changes in cognition or cortical thickness in both the Aβ (+) and Aβ (−) groups (Table 5 and Fig. 2). Despite the proposed ability of leptin to enhance cognition [4] including its anti-Aβ properties shown in animal studies [19], human studies assessing the relation between blood leptin and brain status have shown mixed results [5, 7, 10, 14–17]. Since leptin is mainly secreted from adipose tissue, blood levels of leptin increase with women and higher insulin resistance [38], all of which are associated with an elevated risk of AD. Therefore, the advantageous effect of leptin on cognition might be blurred by these factors in this study, and the predictive potential of plasma leptin on cognition might be weaker than that of adiponectin. The association of plasma leptin level and longitudinal cognition did not differ by sex in our study sample, suggesting that other cardiovascular or metabolic risk factors might affect the relationship between leptin and cognition. Further longitudinal studies with a large sample size are needed to reveal the relationship between leptin and cognition, including consideration of the effect of sex. Noteworthy in our study is that the association between plasma leptin and longitudinal cognition and cortical thickness might not vary by Aβ states.

The strength of this study is that, to the best of our knowledge, it is the first human study to investigate the longitudinal influence of blood adipokines on cognition and brain structure stratified by Aβ status. Furthermore, we used only data from MCI participants without obesity to strengthen the homogeneity of the study sample by excluding the potential confounding effects of obesity on adiponectin or leptin levels and cognition.

This study has also several limitations. First, the levels of different isoforms of adiponectin (trimer, hexamer, and high-molecular-weight form) were not separately measured in this study. Therefore, the relation between cognitive decline and adiponectin multimerization according to Aβ is needed to be assessed in the following studies. Nevertheless, it might be worth noting that the level of blood total adiponectin is strongly associated with that of blood high-molecular-weight adiponectin [39], and also with that of CSF total adiponectin [13]. Second, the information on sarcopenia and central adiposity, which are also known to be associated with blood adiponectin [37, 40], was not available in this study. Data about appendicular skeletal muscle mass, waist circumference, or abdominal fat mass, as well as BMI, may help to elaborate the relationship between adipokines, nutritional status, and cognitive decline. Third, the sample size was relatively small compared with other large population-based cohort studies [5, 6], as we selected participants with CSF analysis data. Fourth, most participants were of European descent, and hence, further studies in other ethnic groups are needed to generalize our findings. Fourth, other brain regions in the medial temporal lobe, such as the hippocampus, dentate gyrus, and perirhinal cortex, were not explored owing to unavailability. However, the thickness of the PHC and ERC is also known to be closely associated with AD-related cognitive changes [24, 25]. Last, it should be noted that some of our interpretations of the findings are highly speculative as our study was observational in nature. Mechanistic (molecular or interventional) studies can finally provide insight into the causal relationship between adipokine (adiponectin or leptin) and cognition.

## Conclusions

In conclusion, the significant association of plasma adiponectin levels with longitudinal changes in cognition and brain structure was demonstrated only in participants with Aβ (+) status, suggesting an interaction between adiponectin and Aβ. In the Aβ (+) condition, higher plasma adiponectin levels at baseline predicted the faster cognitive decline and cortical thinning. In contrast to adiponectin, plasma leptin levels did not predict cognitive decline or cortical thinning either in participants with Aβ (+) or in those with Aβ (−) status. Our findings suggest the potential predictive value of plasma adiponectin for neurodegeneration under Aβ pathology.

## Supplementary Information

**Additional file 1: Fig. S1.** Selection of the study population. **Table S1.** Number of participants who performed ADAS-Cog or MRI scan at each time points. **Table S2.** The number of fully withdrawn participants and the reasons for withdrawal. **Table S3.** Linear mixed-effect model parameter estimates for the association between plasma adiponectin levels and clinical outcomes. **Table S4.** Predictive effect of adiponectin after excluding the PPARγ agonist user. **Table S5.** Linear mixed-effect model parameter estimates for the association between plasma leptin levels and clinical outcomes. **Table S6.** Effect of sex on the association between baseline plasma adipokine levels and longitudinal cognition.

## Data Availability

The data used in this study are from the ADNI database (http://adni.loni.usc.edu) which are accessible to interested scientists with the ADNI Data Use Agreement (http://adni.loni.usc.edu/wp-content/uploads/how_to_apply/ADNI_Data_Use_Agreement.pdf).
