# Supplementary material for: Plasma adiponectin levels predict cognitive decline and cortical thinning in mild cognitive impairment with beta-amyloid pathology
Source: Alzheimers Res Ther. 2022 Nov 4;14:165. doi: 10.1186/s13195-022-01107-3 (PMC9635143; doi:10.1186/s13195-022-01107-3)
Supplement: Supplementary file 1 — Additional file 1: Fig. S1. Selection of the study population. Table S1. Number of participants who performed ADAS-Cog or MRI scan at each time points. Table S2. The number of fully withdrawn participants and the reasons for withdrawal. Table S3. Linear mixed-effect model parameter estimates for the association between plasma adiponectin levels and clinical outcomes. Table S4. Predictive effect of adiponectin after excluding the PPARγ agonist user. Table S5. Linear mixed-effect model parameter estimates for the association between plasma leptin levels and clinical outcomes. Table S6. Effect of sex on the association between baseline plasma adipokine levels and longitudinal cognition. [file 13195_2022_1107_MOESM1_ESM.docx]

**Supplementary figure and tables**

**Supplementary Figure 1. Selection of the study population**

**
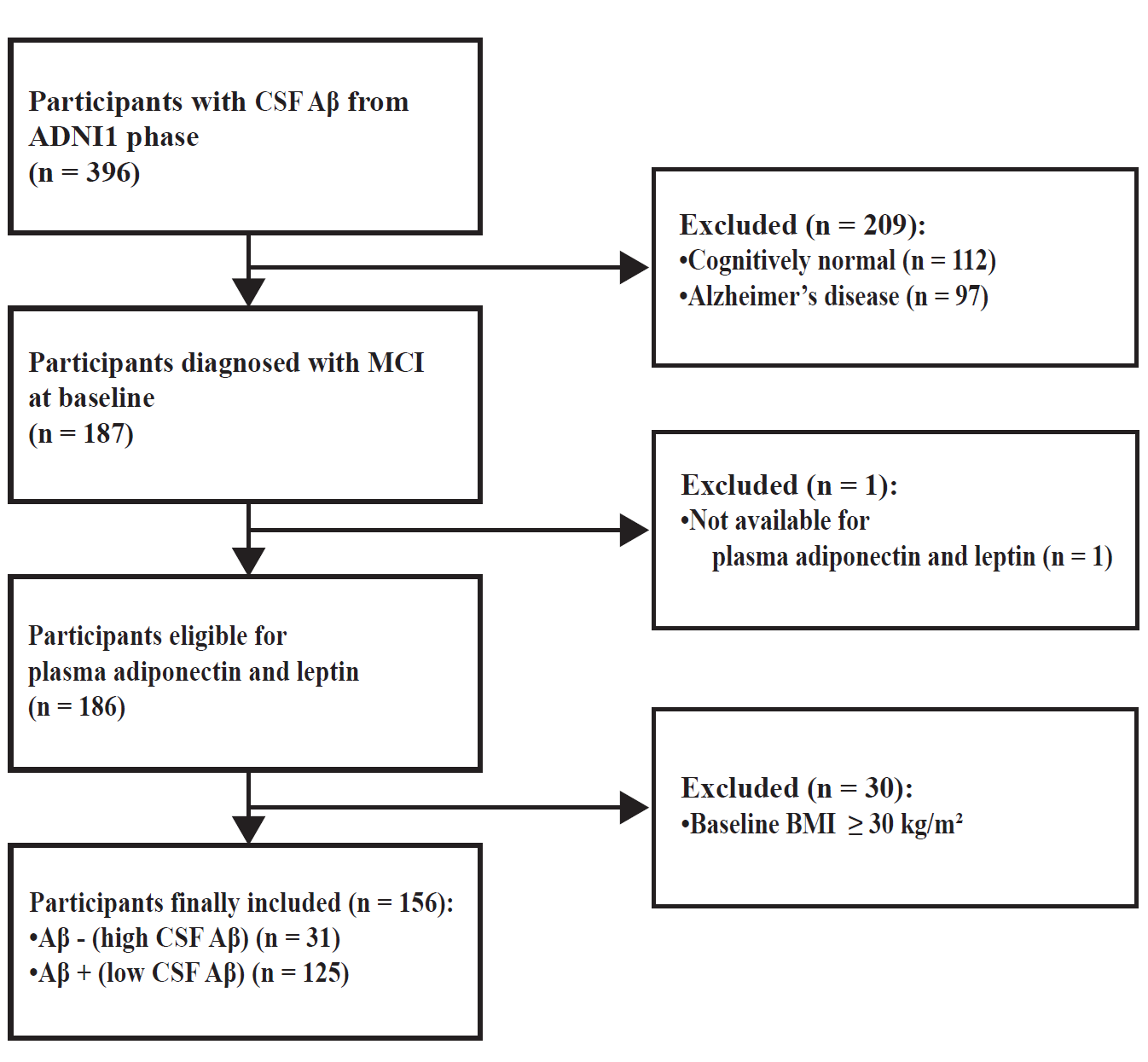
**

Abbreviations: Aβ, amyloid-β; BMI, body mass index; CSF, cerebrospinal fluid; MCI, mild cognitive impairment.

| **Supplementary Table 1. Number of participants who performed ADAS-Cog or MRI scan at each time points** | | | | | |
| --- | --- | --- | --- | --- | --- |
|  | ADAS-Cog | |  | MRI scan | |
| Time | Aβ (-) | Aβ (+) |  | Aβ (-) | Aβ (+) |
| Baseline | 31 | 125 |  | 31 | 125 |
| 6 months | 30 | 118 |  | 31 | 112 |
| 12 months | 29 | 116 |  | 27 | 109 |
| 18 months | 27 | 108 |  | 24 | 99 |
| 24 months | 23 | 103 |  | 21 | 91 |
| 36 months | 21 | 78 |  | 14 | 62 |
| 48 months | 12 | 36 |  | 6 | 33 |
| 60 months | 8 | 34 |  | 5 | 22 |
| 72 months | 10 | 31 |  | 4 | 15 |
| 84 months | 9 | 20 |  |  |  |
| 96 months | 8 | 16 |  |  |  |
| 108 months | 5 | 9 |  |  |  |
| 120 months | 2 | 6 |  |  |  |
| 132 months | 1 | 3 |  |  |  |
| 144 months | 2 | 2 |  |  |  |
| 156 months | 1 |  |  |  |  |
| Abbreviations: Aβ, amyloid-β; ADAS-Cog, Alzheimer's disease assessment scale-cognitive subscale; MRI, magnetic resonance imaging. | | | | | |

| **Supplementary Table 2. The number of fully withdrawn participants and the reasons for withdrawal** | | | | | | | | |
| --- | --- | --- | --- | --- | --- | --- | --- | --- |
| Time | number | Consent withdrawn, loss of follow-up, non-compliance, or non-responsive | Death | Moved to nursing facility | Adverse event or safety risk | Study terminated | Protocol violation | Others |
| Baseline | 3 | 2 | 1 |  |  |  |  |  |
| 6 months | 6 | 6 |  |  |  |  |  |  |
| 12 months | 9 | 7 | 2 |  |  |  |  |  |
| 18 months | 6 | 6 |  |  |  |  |  |  |
| 24 months | 11 | 10 | 1 |  |  |  |  |  |
| 30 months | 9 | 5 | 1 |  | 1 | 1 | 1 |  |
| 36 months | 24 | 18 | 3 |  | 2 |  | 1 |  |
| 42 months | 8 | 6 |  |  | 2 |  |  |  |
| 48 months | 11 | 6 | 2 | 1 | 1 |  | 1 |  |
| 54 months | 4 |  | 1 | 1 | 1 |  |  | 1 |
| 60 months | 1 |  | 1 |  |  |  |  |  |
| 66 months | 3 |  | 1 | 1 |  |  |  | 1 |
| 72 months | 2 | 1 | 1 |  |  |  |  |  |
| 78 months | 4 | 3 | 1 |  |  |  |  |  |
| 84 months | 7 | 2 | 1 | 2 |  |  |  | 2 |
| 90 months | 1 | 1 |  |  |  |  |  |  |
| 96 months | 9 | 4 | 1 | 2 |  | 1 |  | 1 |
| 102 months | 1 |  | 1 |  |  |  |  |  |
| 108 months | 11 | 4 | 5 | 1 |  |  |  | 1 |
| 114 months | 2 |  | 2 |  |  |  |  |  |
| 120 months | 4 | 2 |  |  |  |  |  | 2 |
| 126 months | 3 |  | 2 |  |  |  |  | 1 |
| 132 months | 1 |  | 1 |  |  |  |  |  |
| Total | 140 | 83 | 28 | 8 | 7 | 2 | 3 | 9 |

**Supplementary Table 3. Linear mixed-effect model parameter estimates for the association between plasma adiponectin levels and clinical outcomes**

|  | Aβ (+) | | |  | Aβ (-) | | |
| --- | --- | --- | --- | --- | --- | --- | --- |
|  | Outcome - ADAS-Cog | | | | | | |
| Predictor | beta | SE | p-value |  | beta | SE | p-value |
| Intercept | 8.435 | 9.627 | 0.383 |  | 15.760 | 13.304 | 0.253 |
| Adiponectin | 0.844 | 2.158 | 0.696 |  | 3.461 | 2.871 | 0.243 |
| Time | 0.068 | 0.074 | 0.365 |  | 0.047 | 0.039 | 0.248 |
| Age at baseline | 0.063 | 0.069 | 0.358 |  | -0.008 | 0.097 | 0.937 |
| Gender | 0.538 | 1.063 | 0.614 |  | -1.128 | 2.005 | 0.581 |
| Education | -0.075 | 0.165 | 0.652 |  | -0.320 | 0.234 | 0.189 |
| APOE4 | -0.277 | 0.663 | 0.677 |  | -2.668 | 2.004 | 0.200 |
| BMI | -0.138 | 0.203 | 0.497 |  | -0.186 | 0.271 | 0.503 |
| Hypertension | 0.327 | 0.935 | 0.727 |  | -0.050 | 1.468 | 0.973 |
| Diabetes mellitus | 3.802 | 1.413 | 0.008 |  | 2.128 | 2.305 | 0.369 |
| Smoking | 1.920 | 0.980 | 0.053 |  | 2.394 | 1.653 | 0.165 |
| Alcohol | 1.389 | 2.387 | 0.562 |  | 2.284 | 2.590 | 0.390 |
| Insulin | -2.513 | 1.495 | 0.096 |  | 1.869 | 2.750 | 0.506 |
| eGFR | 0.019 | 0.033 | 0.571 |  | -0.005 | 0.051 | 0.921 |
| **Adiponectin × Time** | 0.224 | 0.093 | 0.018 |  | -0.018 | 0.054 | 0.744 |
|  | Outcome - PHC, right | | | | | | |
| Intercept | 3.608 | 0.595 | < 0.001 |  | 4.391 | 1.353 | 0.005 |
| Adiponectin | -0.082 | 0.135 | 0.542 |  | -0.169 | 0.273 | 0.543 |
| Time | -0.001 | 0.001 | 0.397 |  | -0.002 | 0.002 | 0.294 |
| Age at baseline | -0.009 | 0.004 | 0.036 |  | -0.017 | 0.010 | 0.108 |
| Gender | 0.016 | 0.065 | 0.809 |  | 0.328 | 0.203 | 0.124 |
| Education | -0.020 | 0.010 | 0.048 |  | -0.014 | 0.023 | 0.551 |
| APOE4 | -0.051 | 0.041 | 0.217 |  | -0.006 | 0.200 | 0.975 |
| BMI | -0.009 | 0.013 | 0.456 |  | -0.016 | 0.027 | 0.558 |
| Hypertension | 0.018 | 0.058 | 0.750 |  | 0.068 | 0.148 | 0.652 |
| Diabetes mellitus | 0.009 | 0.087 | 0.920 |  | -0.053 | 0.235 | 0.822 |
| Smoking | 0.105 | 0.060 | 0.086 |  | 0.241 | 0.166 | 0.165 |
| Alcohol | -0.329 | 0.143 | 0.023 |  | 0.138 | 0.264 | 0.608 |
| Insulin | 0.179 | 0.093 | 0.056 |  | -0.246 | 0.277 | 0.387 |
| eGFR | 0.000 | 0.002 | 0.868 |  | -0.002 | 0.005 | 0.765 |
| **Adiponectin × Time** | -0.004 | 0.002 | 0.012 |  | -0.002 | 0.002 | 0.529 |
|  | Outcome - PHC, left | | | | | | |
| Intercept | 4.310 | 0.636 | < 0.001 |  | 4.018 | 1.271 | 0.006 |
| Adiponectin | -0.332 | 0.148 | 0.027 |  | -0.533 | 0.264 | 0.058 |
| Time | -0.001 | 0.001 | 0.400 |  | -0.001 | 0.002 | 0.609 |
| Age at baseline | -0.012 | 0.004 | 0.012 |  | -0.007 | 0.009 | 0.491 |
| Gender | 0.012 | 0.070 | 0.862 |  | 0.426 | 0.191 | 0.039 |
| Education | -0.037 | 0.011 | 0.001 |  | -0.039 | 0.022 | 0.094 |
| APOE4 | -0.082 | 0.044 | 0.065 |  | -0.057 | 0.189 | 0.765 |
| BMI | -0.008 | 0.013 | 0.548 |  | -0.010 | 0.026 | 0.704 |
| Hypertension | 0.040 | 0.062 | 0.518 |  | 0.181 | 0.140 | 0.211 |
| Diabetes mellitus | -0.023 | 0.093 | 0.802 |  | -0.034 | 0.221 | 0.879 |
| Smoking | 0.100 | 0.064 | 0.124 |  | 0.171 | 0.157 | 0.291 |
| Alcohol | -0.383 | 0.154 | 0.014 |  | 0.113 | 0.247 | 0.653 |
| Insulin | 0.075 | 0.099 | 0.452 |  | -0.358 | 0.260 | 0.187 |
| eGFR | -0.001 | 0.002 | 0.816 |  | 0.000 | 0.005 | 0.994 |
| **Adiponectin × Time** | -0.004 | 0.002 | 0.025 |  | -0.002 | 0.003 | 0.411 |
|  | Outcome - ERC, right | | | | | | |
| Intercept | 5.246 | 0.938 | < 0.001 |  | 5.396 | 2.043 | 0.017 |
| Adiponectin | 0.058 | 0.210 | 0.785 |  | -0.245 | 0.412 | 0.559 |
| Time | -0.003 | 0.002 | 0.188 |  | -0.009 | 0.004 | 0.013 |
| Age at baseline | -0.013 | 0.007 | 0.057 |  | -0.030 | 0.015 | 0.059 |
| Gender | -0.108 | 0.103 | 0.298 |  | 0.058 | 0.306 | 0.851 |
| Education | -0.034 | 0.016 | 0.038 |  | -0.020 | 0.035 | 0.577 |
| APOE4 | 0.035 | 0.065 | 0.585 |  | -0.175 | 0.302 | 0.570 |
| BMI | -0.015 | 0.020 | 0.448 |  | 0.000 | 0.041 | 0.997 |
| Hypertension | -0.097 | 0.091 | 0.290 |  | 0.066 | 0.224 | 0.770 |
| Diabetes mellitus | -0.133 | 0.137 | 0.336 |  | 0.055 | 0.354 | 0.878 |
| Smoking | 0.052 | 0.095 | 0.583 |  | 0.368 | 0.251 | 0.161 |
| Alcohol | -0.414 | 0.225 | 0.069 |  | 0.033 | 0.398 | 0.936 |
| Insulin | 0.331 | 0.146 | 0.025 |  | -0.169 | 0.419 | 0.691 |
| eGFR | -0.005 | 0.003 | 0.139 |  | 0.006 | 0.008 | 0.418 |
| **Adiponectin × Time** | -0.007 | 0.003 | 0.024 |  | 0.010 | 0.005 | 0.042 |
|  | *continued* | | | | | | |
|  |  |  |  |  |  |  |  |
|  |  |  |  |  |  |  |  |
| Supplementary Table 3, *continued* | | | | | | | |
|  | Aβ (+) | | |  | Aβ (-) | | |
|  | Outcome - ERC, left | | | | | | |
| Predictor | beta | SE | p-value |  | beta | SE | p-value |
| Intercept | 6.124 | 0.988 | < 0.001 |  | 4.792 | 2.258 | 0.048 |
| Adiponectin | -0.061 | 0.222 | 0.785 |  | -0.253 | 0.468 | 0.594 |
| Time | -0.004 | 0.002 | 0.106 |  | -0.004 | 0.003 | 0.245 |
| Age at baseline | -0.023 | 0.007 | 0.002 |  | -0.021 | 0.016 | 0.212 |
| Gender | -0.272 | 0.108 | 0.014 |  | 0.457 | 0.340 | 0.195 |
| Education | -0.035 | 0.017 | 0.041 |  | -0.056 | 0.039 | 0.173 |
| APOE4 | -0.002 | 0.068 | 0.981 |  | -0.190 | 0.335 | 0.576 |
| BMI | -0.026 | 0.021 | 0.209 |  | 0.017 | 0.046 | 0.715 |
| Hypertension | -0.023 | 0.096 | 0.815 |  | 0.100 | 0.249 | 0.692 |
| Diabetes mellitus | -0.003 | 0.145 | 0.983 |  | 0.424 | 0.392 | 0.294 |
| Smoking | 0.079 | 0.100 | 0.434 |  | 0.360 | 0.279 | 0.213 |
| Alcohol | -0.362 | 0.237 | 0.130 |  | 0.134 | 0.440 | 0.765 |
| Insulin | 0.195 | 0.154 | 0.207 |  | -0.570 | 0.464 | 0.235 |
| eGFR | -0.002 | 0.003 | 0.467 |  | 0.003 | 0.009 | 0.695 |
| **Adiponectin × Time** | -0.005 | 0.003 | 0.122 |  | 0.002 | 0.004 | 0.690 |
|  | Outcome - BMI change | | | | | | |
| Intercept | 1.797 | 1.455 | 0.219 |  | 0.665 | 2.388 | 0.784 |
| Adiponectin | 0.083 | 0.325 | 0.799 |  | -0.070 | 0.546 | 0.900 |
| Time | -0.006 | 0.013 | 0.611 |  | -0.036 | 0.031 | 0.257 |
| Age at baseline | -0.002 | 0.011 | 0.866 |  | 0.000 | 0.018 | 0.980 |
| Gender | -0.154 | 0.163 | 0.345 |  | 0.298 | 0.357 | 0.414 |
| Education | -0.058 | 0.025 | 0.022 |  | -0.050 | 0.043 | 0.256 |
| APOE4 | 0.031 | 0.100 | 0.753 |  | 0.037 | 0.361 | 0.920 |
| BMI | -0.042 | 0.031 | 0.178 |  | 0.001 | 0.048 | 0.981 |
| Hypertension | 0.066 | 0.141 | 0.644 |  | -0.318 | 0.265 | 0.245 |
| Diabetes mellitus | -0.137 | 0.216 | 0.526 |  | 0.063 | 0.411 | 0.880 |
| Smoking | 0.084 | 0.150 | 0.574 |  | 0.009 | 0.292 | 0.977 |
| Alcohol | -0.240 | 0.380 | 0.528 |  | 0.269 | 0.470 | 0.573 |
| Insulin | 0.205 | 0.226 | 0.366 |  | 0.651 | 0.489 | 0.199 |
| eGFR | 0.003 | 0.005 | 0.521 |  | 0.000 | 0.009 | 0.997 |
| **Adiponectin × Time** | -0.004 | 0.016 | 0.805 |  | 0.054 | 0.042 | 0.216 |
| The levels of plasma adiponectin and insulin were log transformed. Abbreviations: Aβ, amyloid-β; ADAS-Cog, Alzheimer's Disease Assessment Scale-Cognitive subscale; APOE, apolipoprotein E; BMI, body mass index; ERC, entorhinal cortex; eGFR, estimated glomerular filtration rate; PHC, parahippocampal cortex; SE, standard error. | | | | | | | |

**Supplementary Table 4. Predictive effect of adiponectin after excluding the PPARγ agonist user**

|  | Adiponectin × time interaction | | | | | | |
| --- | --- | --- | --- | --- | --- | --- | --- |
|  | Aβ (+) | | |  | Aβ (-) | | |
| Outcome | beta | SE | p-value |  | beta | SE | p-value |
| ADAS-Cog | -0.004 | 0.002 | 0.029 |  | -0.018 | 0.054 | 0.744 |
| PHC, right | -0.004 | 0.002 | 0.016 |  | -0.002 | 0.002 | 0.529 |
| PHC, left | 0.226 | 0.093 | 0.018 |  | -0.002 | 0.003 | 0.411 |
| ERC, right | -0.007 | 0.003 | 0.026 |  | 0.010 | 0.005 | 0.042 |
| ERC, left | -0.005 | 0.003 | 0.134 |  | 0.002 | 0.004 | 0.690 |
| BMI change | -0.003 | 0.016 | 0.828 |  | 0.054 | 0.042 | 0.216 |
| Models were adjusted for following covariates: baseline age, sex, years of education, number of APOE ε4 genotype, BMI, history of hypertension, diabetes mellitus, smoking and alcohol abuse, the levels of plasma insulin, and eGFR. The levels of plasma adiponectin and insulin levels were log transformed. Abbreviations: Aβ, amyloid-β; ADAS-Cog, Alzheimer's Disease Assessment Scale-Cognitive subscale; APOE, apolipoprotein E; BMI, body mass index; ERC, entorhinal cortex; eGFR, estimated glomerular filtration rate; PHC, parahippocampal cortex; PPARγ, peroxisome proliferator-activated receptor γ; SE, standard error. | | | | | | | |

**Supplementary Table 5. Linear mixed-effect model parameter estimates for the association between plasma leptin levels and clinical outcomes**

|  | Aβ (+) | | |  | Aβ (-) | | |
| --- | --- | --- | --- | --- | --- | --- | --- |
|  | Outcome - ADAS-Cog | | | | | | |
| Predictor | beta | SE | p-value |  | beta | SE | p-value |
| Intercept | 11.765 | 9.413 | 0.214 |  | 19.277 | 13.511 | 0.172 |
| Leptin | 2.689 | 1.864 | 0.152 |  | 0.509 | 2.338 | 0.830 |
| Time | 0.163 | 0.054 | 0.003 |  | 0.036 | 0.030 | 0.248 |
| Age at baseline | 0.065 | 0.069 | 0.344 |  | -0.002 | 0.102 | 0.982 |
| Gender | -0.676 | 1.358 | 0.620 |  | -0.386 | 2.183 | 0.862 |
| Education | -0.060 | 0.164 | 0.718 |  | -0.430 | 0.224 | 0.072 |
| APOE4 | -0.203 | 0.663 | 0.760 |  | -2.498 | 2.130 | 0.256 |
| BMI | -0.342 | 0.233 | 0.145 |  | -0.208 | 0.316 | 0.519 |
| Hypertension | 0.026 | 0.958 | 0.979 |  | -0.268 | 1.507 | 0.861 |
| Diabetes mellitus | 4.137 | 1.428 | 0.005 |  | 2.710 | 2.362 | 0.267 |
| Smoking | 1.738 | 0.977 | 0.078 |  | 2.826 | 1.642 | 0.103 |
| Alcohol | 1.428 | 2.388 | 0.551 |  | 3.284 | 2.590 | 0.222 |
| Insulin | -3.299 | 1.585 | 0.040 |  | -0.487 | 2.516 | 0.849 |
| eGFR | 0.025 | 0.033 | 0.450 |  | 0.005 | 0.053 | 0.926 |
| **Leptin × Time** | 0.091 | 0.059 | 0.128 |  | 0.000 | 0.030 | 0.988 |
|  | Outcome - PHC, right | | | | | | |
| Intercept | 3.363 | 0.582 | < 0.001 |  | 4.164 | 1.281 | 0.004 |
| Leptin | -0.132 | 0.116 | 0.258 |  | 0.306 | 0.214 | 0.169 |
| Time | -0.005 | 0.001 | < 0.001 |  | -0.004 | 0.001 | < 0.001 |
| Age at baseline | -0.009 | 0.004 | 0.032 |  | -0.014 | 0.010 | 0.175 |
| Gender | 0.066 | 0.084 | 0.430 |  | 0.151 | 0.206 | 0.472 |
| Education | -0.022 | 0.010 | 0.035 |  | -0.010 | 0.021 | 0.648 |
| APOE4 | -0.054 | 0.041 | 0.194 |  | 0.073 | 0.199 | 0.719 |
| BMI | 0.004 | 0.014 | 0.794 |  | -0.036 | 0.030 | 0.242 |
| Hypertension | 0.033 | 0.059 | 0.578 |  | 0.093 | 0.142 | 0.522 |
| Diabetes mellitus | -0.011 | 0.088 | 0.897 |  | -0.045 | 0.223 | 0.844 |
| Smoking | 0.106 | 0.060 | 0.080 |  | 0.220 | 0.153 | 0.167 |
| Alcohol | -0.335 | 0.143 | 0.021 |  | 0.105 | 0.246 | 0.674 |
| Insulin | 0.222 | 0.098 | 0.026 |  | -0.254 | 0.236 | 0.296 |
| eGFR | -0.001 | 0.002 | 0.709 |  | 0.000 | 0.005 | 0.943 |
| **Leptin × Time** | 0.001 | 0.001 | 0.485 |  | 0.001 | 0.001 | 0.190 |
|  | Outcome - PHC, left | | | | | | |
| Intercept | 3.800 | 0.648 | < 0.001 |  | 3.285 | 1.392 | 0.030 |
| Leptin | -0.058 | 0.131 | 0.659 |  | 0.123 | 0.233 | 0.604 |
| Time | -0.005 | 0.001 | < 0.001 |  | -0.003 | 0.001 | 0.053 |
| Age at baseline | -0.012 | 0.005 | 0.013 |  | -0.004 | 0.010 | 0.680 |
| Gender | -0.013 | 0.093 | 0.893 |  | 0.221 | 0.223 | 0.334 |
| Education | -0.041 | 0.011 | < 0.001 |  | -0.015 | 0.023 | 0.518 |
| APOE4 | -0.084 | 0.046 | 0.069 |  | 0.027 | 0.216 | 0.900 |
| BMI | 0.009 | 0.016 | 0.594 |  | -0.019 | 0.032 | 0.571 |
| Hypertension | 0.049 | 0.066 | 0.463 |  | 0.225 | 0.154 | 0.163 |
| Diabetes mellitus | -0.053 | 0.098 | 0.590 |  | -0.100 | 0.243 | 0.684 |
| Smoking | 0.068 | 0.067 | 0.311 |  | 0.068 | 0.166 | 0.687 |
| Alcohol | -0.387 | 0.160 | 0.017 |  | -0.076 | 0.267 | 0.779 |
| Insulin | 0.117 | 0.110 | 0.290 |  | -0.074 | 0.257 | 0.777 |
| eGFR | -0.001 | 0.002 | 0.769 |  | -0.001 | 0.005 | 0.863 |
| **Leptin × Time** | 0.001 | 0.001 | 0.428 |  | 0.000 | 0.001 | 0.774 |
|  | Outcome - ERC, right | | | | | | |
| Intercept | 5.037 | 0.908 | < 0.001 |  | 5.154 | 2.010 | 0.020 |
| Leptin | -0.272 | 0.180 | 0.135 |  | -0.332 | 0.336 | 0.337 |
| Time | -0.008 | 0.002 | < 0.001 |  | -0.002 | 0.002 | 0.352 |
| Age at baseline | -0.013 | 0.007 | 0.058 |  | -0.033 | 0.015 | 0.043 |
| Gender | 0.036 | 0.131 | 0.781 |  | 0.176 | 0.323 | 0.593 |
| Education | -0.034 | 0.016 | 0.036 |  | -0.013 | 0.033 | 0.705 |
| APOE4 | 0.032 | 0.064 | 0.620 |  | -0.266 | 0.312 | 0.406 |
| BMI | 0.002 | 0.022 | 0.947 |  | 0.022 | 0.047 | 0.636 |
| Hypertension | -0.070 | 0.092 | 0.453 |  | 0.041 | 0.223 | 0.857 |
| Diabetes mellitus | -0.165 | 0.137 | 0.233 |  | -0.018 | 0.350 | 0.960 |
| Smoking | 0.083 | 0.094 | 0.377 |  | 0.303 | 0.240 | 0.224 |
| Alcohol | -0.410 | 0.223 | 0.069 |  | -0.001 | 0.386 | 0.998 |
| Insulin | 0.403 | 0.153 | 0.010 |  | 0.054 | 0.370 | 0.886 |
| eGFR | -0.005 | 0.003 | 0.104 |  | 0.004 | 0.008 | 0.588 |
| **Leptin × Time** | -0.001 | 0.002 | 0.723 |  | 0.000 | 0.002 | 0.998 |
|  | *continued* | | | | | | |
|  |  |  |  |  |  |  |  |
|  |  |  |  |  |  |  |  |
| Supplementary Table 5, *continued* | | | | | | | |
|  | Aβ (+) | | |  | Aβ (-) | | |
|  | Outcome - ERC, left | | | | | | |
| Predictor | beta | SE | p-value |  | beta | SE | p-value |
| Intercept | 5.765 | 0.961 | < 0.001 |  | 4.603 | 2.245 | 0.055 |
| Leptin | -0.225 | 0.191 | 0.243 |  | 0.103 | 0.381 | 0.790 |
| Time | -0.007 | 0.002 | < 0.001 |  | -0.003 | 0.002 | 0.116 |
| Age at baseline | -0.022 | 0.007 | 0.002 |  | -0.020 | 0.017 | 0.243 |
| Gender | -0.173 | 0.138 | 0.213 |  | 0.341 | 0.362 | 0.359 |
| Education | -0.037 | 0.017 | 0.032 |  | -0.049 | 0.037 | 0.203 |
| APOE4 | -0.006 | 0.068 | 0.924 |  | -0.137 | 0.350 | 0.699 |
| BMI | -0.007 | 0.024 | 0.771 |  | 0.008 | 0.052 | 0.884 |
| Hypertension | 0.004 | 0.098 | 0.968 |  | 0.129 | 0.250 | 0.611 |
| Diabetes mellitus | -0.035 | 0.145 | 0.811 |  | 0.395 | 0.391 | 0.325 |
| Smoking | 0.089 | 0.099 | 0.371 |  | 0.324 | 0.269 | 0.244 |
| Alcohol | -0.363 | 0.236 | 0.127 |  | 0.073 | 0.430 | 0.867 |
| Insulin | 0.272 | 0.163 | 0.097 |  | -0.484 | 0.415 | 0.259 |
| eGFR | -0.003 | 0.003 | 0.394 |  | 0.003 | 0.009 | 0.719 |
| **Leptin × Time** | 0.000 | 0.002 | 0.833 |  | 0.000 | 0.002 | 0.890 |
| The levels of plasma leptin and insulin were log transformed. Abbreviations: Aβ, amyloid-β; ADAS-Cog, Alzheimer's Disease Assessment Scale-Cognitive subscale; APOE, apolipoprotein E; BMI, body mass index; ERC, entorhinal cortex; eGFR, estimated glomerular filtration rate; PHC, parahippocampal cortex; SE, standard error. | | | | | | | |

| **Supplementary Table 6. Effect of sex on the association between baseline plasma adipokine levels and longitudinal cognition** | | | | | | | | |
| --- | --- | --- | --- | --- | --- | --- | --- | --- |
|  |  | Adiponectin × time interaction | | | | | | |
|  |  | Aβ (+) | | |  | Aβ (-) | | |
| Outcome |  | beta | SE | p-value |  | beta | SE | p-value |
| ADAS-Cog | Male | 0.170 | 0.102 | 0.102 |  | -0.035 | 0.078 | 0.663 |
|  | Female | 0.054 | 0.179 | 0.763 |  | 0.106 | 0.074 | 0.181 |
|  |  | Leptin × time interaction | | | | | | |
| ADAS-Cog | Male | -0.058 | 0.064 | 0.377 |  | 0.029 | 0.052 | 0.593 |
|  | Female | 0.118 | 0.132 | 0.379 |  | -0.013 | 0.044 | 0.784 |
|  |  | Adiponectin × time × sex interaction | | | | | | |
| ADAS-Cog |  | -0.132 | 0.188 | 0.485 |  | 0.131 | 0.129 | 0.321 |
|  |  | Leptin × time × sex interaction | | | | | | |
| ADAS-Cog |  | 0.182 | 0.131 | 0.170 |  | -0.043 | 0.088 | 0.640 |
| Models were adjusted for following covariates: baseline age, sex, years of education, number of APOE ε4 genotype, BMI, history of hypertension, diabetes mellitus, smoking and alcohol abuse, the levels of plasma insulin, and eGFR. The levels of plasma adiponectin, leptin, and insulin were log transformed. Abbreviations: Aβ, amyloid-β; ADAS-Cog, Alzheimer's Disease Assessment Scale-Cognitive subscale; APOE, apolipoprotein E; BMI, body mass index; eGFR, estimated glomerular filtration rate; SE, standard error. | | | | | | | | |
